# Presentation of Complex Homozygous Allele in *ABCA4* Gene in a Patient with Retinitis Pigmentosa

**DOI:** 10.1155/2015/452068

**Published:** 2015-07-02

**Authors:** Māreta Audere, Katrīna Rutka, Svetlana Šepetiene, Baiba Lāce

**Affiliations:** ^1^Biomedical Research and Study Centre, Riga LV-1067, Latvia; ^2^Pauls Stradins Clinical University Hospital, Riga LV-1002, Latvia

## Abstract

Retinitis pigmentosa is a degenerative retinal disease characterized by progressive photoreceptor damage, which causes loss of peripheral and night vision and the development of tunnel vision and may result in loss of central vision. This study describes a patient with retinitis pigmentosa caused by a mutation in the *ABCA4* gene with complex allele c.1622T>C, p.L541P; c.3113C>T, p.A1038V in homozygous state.

## 1. Introduction

Retinitis pigmentosa (RP) is a degenerative retinal disease characterized by progressive photoreceptor damage, which causes loss of peripheral and night vision, the development of tunnel vision and may result in loss of central vision. Changes in fundus oculi include specific lumps of pigment called “bone corpuscles.” The phenotype of RP is very diverse, with the same mutation resulting in various clinical presentations, as well as differences in age at onset, severity, and rate of progression [1]. Worldwide the prevalence of RP is approximately 1 in 5,000 individuals [2]. RP is frequently associated with posterior subcapsular cataracts, myopia, astigmatism, keratoconus, and mild hearing loss, not including those with the Usher syndrome, who have RP and hearing impairment.

RP includes a group of inherited disorders, with several patterns of inheritance, although in 50–60% of patients RP is inherited in an autosomal recessive manner [3]. Mutations in approximately 70 genes have been shown to result in autosomal recessive inheritance of RP. In about 30–40% of patients, RP is inherited as an autosomal dominant disorder, with 5–15% having X-linked disease [3]. In addition, a syndromic mitochondrial form, previously called RP8 and RP21 and caused by a mutation in* MTTS2* gene, and a digenic diallelic form, involving two genes,* RDS* and* ROM1*, have been described [4, 5].

The diagnosis of RP is rather challenging because of the many possible genes. More than 20 genes are reported to cause the autosomal dominant type of RP, with the* RHO* gene being the most frequent (20–30%). The autosomal recessive type of RP can be caused by at least 35 genes, with mutations in the* USH2A* gene being the most frequent (10–15%). Most cases of X-linked RP are caused by mutations in* RPGR* and* RP2* [6, 7]. A hierarchical approach to the efficient genetic diagnosis of the autosomal dominant type of RP uses 40 di-, tri-, and tetranucleotide repeats, associated with 10 genes responsible for autosomal dominant RP, as markers in multiplex amplification and genotyping, followed by linkage analysis [8]. This allowed the exclusion of the most possible gene candidates, because of the discordance of cosegregation. Subsequently, the remaining genes were screened for mutations. This systematic approach facilitated the molecular diagnosis of autosomal dominant RP [8]. This study describes a patient with RP caused by a mutation in the* ABCA4* gene.

## 2. Case Presentation

**Figure 1:**
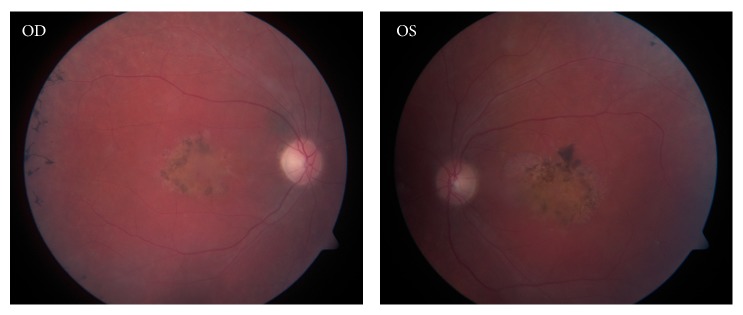
Fundus oculi examination of proband.

The proband (III-2) was a 27-year-old woman who first complained about vision at the age of 8 years. Fundoscopic examination at that time revealed retinal dystrophy. Audiometric testing showed normal hearing. In 2005, at the age of 17 years, the patient had low visual acuity, 20/400, in both eyes. The intraocular pressure (IOP) was 18 mm Hg in her right eye (OD) and 20 mm Hg in her left eye (OS). Optic coherence tomography (OCT) showed decreased thickness of the retinal layers in both eyes. Fundus oculi examination showed focal pathology in the macular region and “bone corpuscle” like pigment lumps in the periphery (Figure 1). Visual field testing in 2006 showed noticeable tunnel-like vision changes. Ocular examinations in 2008 and 2011 showed no further changes in her clinical status. An electroretinogram (ERG) and visually evoked potential (VEP) in 2012 both showed explicit changes in the a- and b-wave amplitudes of both eyes. ERG showed that in both eyes the a- and b-waves had very insufficient amplitudes and inadequate implicit times. The VEP showed drastically decreased N75-P100 amplitude in both eyes and prolonged P100 implicit time in the right eye.

**Figure 2:**
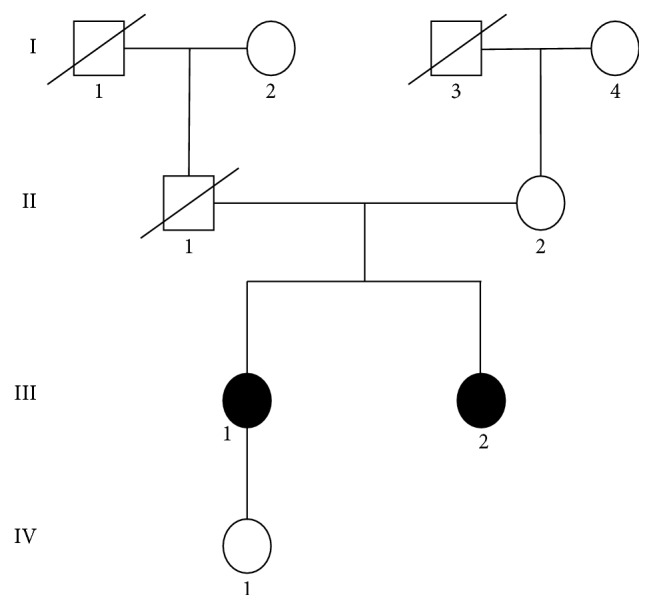
Family tree of patient.

The sister of the proband showed a similar clinical presentation at 8 years of age. Their parents were not related and came from different nationalities. The family tree is represented in Figure 2.

DNA diagnostics were performed in 2013. The* ABCA4*,* ARL6*,* C2ORF71*,* CNGB1*,* CRB1*,* EYS*,* IDH3B*,* IMPG2*,* MERTK*,* NR2E3*,* PDE6A*,* PDE6B*,* PDE6G*,* PRCD*,* PROM1*,* RDH12*,* RGR*,* RHO*,* RP1*,* RPE65*,* SAG*,* SEMA4A*,* TTC8*,* TULP1*,* USH2A*, and* ZNF513* genes were analyzed by PCR and next-generation sequencing of both DNA strands of their entire coding regions and their highly conserved exon-intron splice junctions. PCR-based amplicon library capture was utilized, with a minimum coverage of 30x for each amplicon. Mutations were observed in three genes: (1) homozygous mutations in exons 12 and 21 of the* ABCA4* gene (c.1622T>C, p.L541P; c.3113C>T, p.A1038V), (2) a heterozygous variant in exon 2 of the* NR2E3* gene (c.227G>A, p.R760), and (3) a heterozygous variant in exon 6 of the* RGR* gene (c.747A>T p.K249N). Based on previous knowledge of these three genes, it was concluded that the* ABCA4* mutations (c.1622T>C, p.L541P; c.3113C>T, p.A1038V) were those that likely caused RP in this patient.

## 3. Discussion

*ABCA4* gene mutations are considered among the most common mutations causing RP, cone rod dystrophy, and Stargardt disease [9]. Two disease-causing alleles in the* ABCA4* gene, L541P (c.1622T>C, p.Leu541Pro, rs61751392) and A1038V (c.3113C>T, p.Ala1038Val, rs61751374), were identified in cis-configuration, with this co-occurance later designating the complex allele L541P;A1038V [10]. This complex occurs more frequently in people of German descent and is therefore regarded as a German founder mutation. Indeed, 12.7% of patients with Stargardt disease are from Germany, compared with 1.1% in non-German populations [11, 12].

To date, however, persons homozygous for this complex allele have been described in only a few families. Their age of RP onset is quite young, with the youngest reported to be 3 years at disease onset [13]. Both our proband and her sister were 8 years old at diagnosis of RP, the same age as that in a family with RP associated with the L541P;A1038V complex [14]. In contrast, the age of onset of Stargardt disease is usually from 10 to 14 years [11]. Segregation analysis for family was not performed, which has certain limitation to the conclusions. Novel mutations in the genes* NR2E3* and* RGR* could be related to retinitis pigmentosa and affect the phenotype; still these were not likely to be the main causative genes.

Patients homozygous for the L541P;A1038V complex allele have been found to present with various clinical symptoms, including RP, cone rod dystrophy, age related macular degeneration, and Stargardt disease. For example, a 35-year-old white female of Polish German ancestry homozygous for the complex allele L541P;A1038V was found to have cone rod dystrophy [15]. Her visual acuity was OD 10/350 and OS 5/400, and central and peripheral vision field losses were observed, ERG showed equal reductions in numbers of cones and rods, and fundus oculi findings indicated diffuse pigmentary degenerative changes.

Another report described a family with two patients having classic RP symptoms [14]. Macular involvement was observed in both retinas of both patients. The younger sibling had a visual acuity of CF 3 feet (OD) and CF 2 feet (OS), whereas the older sibling had a visual acuity of CF 5 feet (OD) and HM OS.

Another case report described a patient with Stargardt disease and a severe phenotype. His visual acuity was 1.2 bilaterally. Fundoscopy showed atrophic-appearing foveal and macular lesions and numerous diffuse yellowish deposits. Electroretinography revealed light-adapted 3.0 cd × s × m 230 Hz flicker, but single flash cone and scotopic bright flash were negative [13].

The patient described in this case report has very low vision, with a bilateral visual acuity of 20/400. A fundus oculi examination showed macular focal pathology and peripheral pigmentary degenerative changes. Tunnel-like vision fields were observed. Since the age of 17 years, however, disease progression has not been observed.

Homozygosity of the L541P;A1038V complex allele causes a severe phenotype, characterized by early presentation and retinal and macular involvement. This clinical picture is thought to be due to protein mislocalization. Using the frog eye model, proteins encoded by the* ABCA4* gene containing mutations were not found to be processed properly through the Golgi and endoplasmic reticulum. Aberrant processing is a feature of misfolded proteins, thought to be due mostly to the L541P mutation. Although this may explain the early disease onset, it did not explain variations in retinal phenotype [14].
